# Angioleiomyoma of the proper ulnar digital artery: Case report

**DOI:** 10.1016/j.ijscr.2020.04.013

**Published:** 2020-05-08

**Authors:** Abdullah Essam Kattan, Khalid Arab, Mohammed Abdullah Alswayyed, Emran Algadiem, Qutaiba N.M. Shah Mardan

**Affiliations:** a King Saud University, College of Medicine, Department of Surgery, Division of Plastic Surgery, Riyadh, King Khalid Road. P.O. Box 7805, 12372, Saudi Arabia; b King Saud University, College of Medicine, Department of Pathology and Laboratory Medicine, Riyadh, King Khalid Road. P.O. Box 7805, 12372, Saudi Arabia; c King Fahad Hospital, Department of Surgery, Division of Plastic Surgery, Eastern Province, Hofuf, Prince Salman Street, 36441, Saudi Arabia; d Sulaiman Al Rajhi Colleges, College of Medicine, Al-Qassim, Al-Bukariya, Muhammad Ibn Ali Al Suwaylim Street, P.O. Box 777, 51941, Saudi Arabia

**Keywords:** PD, Proton density, Angioleiomyoma, Hand tumor, Case report

## Abstract

**Introduction:**

With the rarity and benign nature, angioleiomyoma is a tumor of disputed etiology that originates from the tunica media of the blood vessels. Lacking any characteristic clinical or radiological features, often leading to preoperative misdiagnosis, it is only diagnosed confidently on the basis of histopathology; for this reason combined with the rarity, angioleiomyoma would not be usually found at the top of the differential diagnosis list. Local excision is usually curative with excellent prognosis.

**Case:**

This report discusses a case of a 60-year-old healthy lady who complained of a chronic painless mass in the right 2nd digit. Originating from the proper ulnar digital artery, it was excised with no complications. Histopathology results confirmed the diagnosis of angioleiomyoma. No recurrence was observed 6 months following the procedure.

**Discussion:**

Out of the handful published papers concerned with angioleiomyoma in hands, few report the origin to be the digital artery.

**Conclusion:**

Further awareness about this tumor is required and it should be included in the differential diagnoses list of the treating surgeon.

## Highlights

• Angioleiomyoma is a rare benign tumor comprised of smooth muscle cells.
• Other than biopsy, no modality of investigation can conclude the diagnosis.
• Favorable prognosis can be obtained by simple local excision.
• This case report presents a condition in which this tumor emerged from hand’s artery - An extraordinary observation.

## 1 Introduction

Initially described in 1937, angioleiomyoma, also known as angiomyoma, vascular leiomyoma or dermal angioma, is a benign tumor composed of smooth muscle bundles that arise from the tunica media of the subcutaneous blood vessels [1,2]. The hands are devoid of smooth muscle cells, except for the tunica media of the blood vessels, henceforth the rarity of this tumor in the hands [3]. Comprising 5% of all soft tissue tumors, any body part is liable to develop angioleiomyoma, particularly the lower limbs. Surgical excision of the lesion is the mainstay of management with an overall good prognosis. Extremely rare cases of malignant transformation have been documented [4,5]. The current literature is limited to case reports and series as a result of scarcity of this condition in the upper extremities, especially the digits; thus, many steps lie ahead to attain a full picture of this disease. Making it more extraordinary, a handful of case reports were published documenting the tumor to have arisen from the digital artery. In concordance with SCARE guidelines [6], herein is a case of angioleiomyoma of the right index that originated from the ulnar digital artery.

## 2 Case

**Fig. 1:**
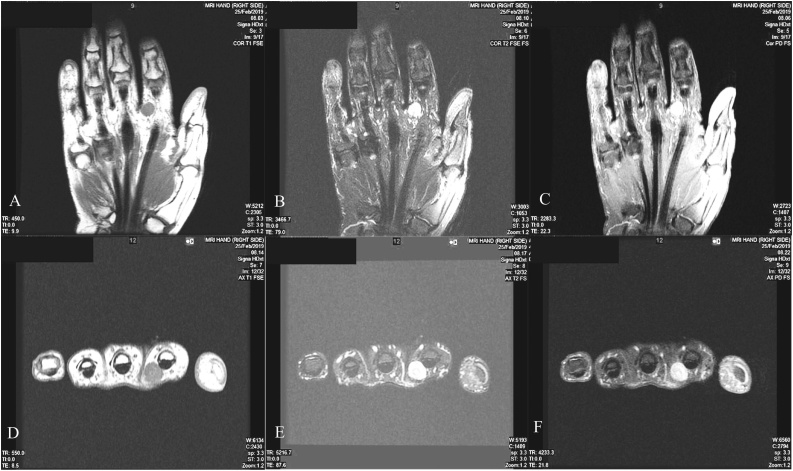
MRI of the right hand taken on different views and modes showing the mass. A: Coronal T1; B: Coronal T2; C: Coronal PD; D: Axial T1; E: Axial T2; F: Axial PD.

**Fig. 2:**
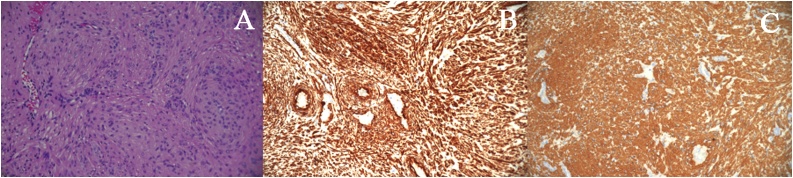
Light microscopy photograph of the lesion. A: Smooth muscle cells surrounding vascular lumina (Hematoxylin and eosin stain); B: Cytoplasmic positivity to smooth muscle actin (SMA) on immunohistochemistry staining; C: Cytoplasmic positivity to caldesmon on immunohistochemistry staining. (All magnifications are ×200).

**Fig. 3:**
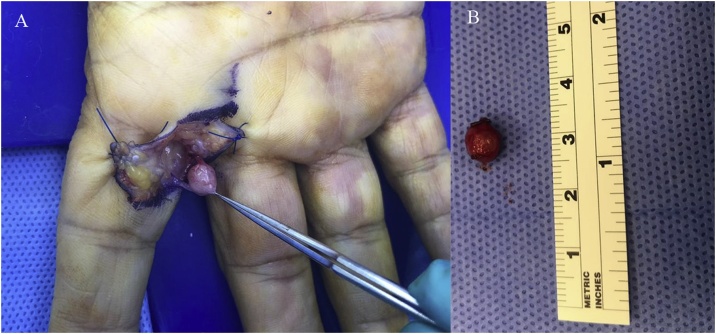
A: Intraoperative picture of the lesion; B: The lesion after excision.

A 60-year-old right-handed lady, not known to have any medical conditions, presented to the plastic surgery clinic at our institute complaining of a painless mass on the right index finger for a long time. No history of trauma was documented. On examination it was a small, firm mass located on the ulnar base of the right 2nd digit’s proximal phalanx. Magnetic resonance imaging (MRI) showed a soft tissue nodule measuring 1 × 0.8 × 1 cm at the ulnar aspect of the 2nd digit base, close to the palmoulnar side of the flexor tendon. Hypointense signal was evident on T1 while T2 demonstrated inhomogeneous hyperintense signal. PD mode showed an internal hypointense ring with a central hypointense dot; nerve sheath tumor was proposed based on the aforementioned radiological findings. Intraoperatively Bruner incision over the mass was done under general anesthesia followed by dissection and flap creation. The mass was found to sprout from the proper ulnar digital artery and was excised with the branches were clipped; there were no intraoperative or postoperative complications. The patient was sent home on analgesia, antibiotics and dressing. Two weeks following the operation, pathology report concluded the diagnosis of angioleiomyoma. The patient’s wound healed by that time and no pain was reported. No recurrence was reported on a follow-up visit 6 months later (Fig. 1, Fig. 2, Fig. 3).

## 3 Discussion

With a tendency towards females, male to female ratio of 1: 1.7, and those ageing between thirty to sixty years old, angioleiomyoma appears as a slowly growing, firm mass when found in the hands [3]. It is pain-free in approximately 60% of the cases especially when the upper extremity is involved, albeit pain is the most striking feature when present. A distinguishing hallmark is that angioleiomyomas are not typically located beyond the distal interphalangeal joints of the hands [2]. Considered as a subtype of vascular tumors, angioleiomyomas account for less than 1% of all upper extremity soft tissue tumors [1,7]. Although many postulations are presented, the exact etiology of this tumor is yet to be identified. Theories include venous stasis, hormonal alterations especially estrogen and chronic microtrauma [2,3]. Less favorable proposals include a hamartomatous process or smooth muscle proliferation of a hemangioma [1].

Histologically angioleiomyoma is divided to three types: Cavernous, venous and solid, with the last one being the most common in the extremities and more likely to be painful [2,3,8]. Tissue examination demonstrates a thick capsule surrounding smooth muscle bundles and vascular channels that exhibit no signs of cellular atypia or mitotic activity [1]. Presence of nerve fibers prones to painful swellings since they constrict blood vessels leading to painful ischemia [3]. Immunohistochemistry is positive for SMA, CD31 and CD34 [9]. There are many findings using different modalities of imaging. Ultrasound shows defined margins with homogenous structure. Doppler ultrasound demonstrates high resistance in the small arteries traversing the mass. Using T2-weighted MRI, hyperintense and isointense areas over the mass are seen [2]. All thereof findings are not pathognomonic for angioleiomyoma [3].

There are scarce reports about cases of angioleiomyoma in the hands [[1], [2], [3],^10^]. Even rarer are the cases describing the tumor to have emerged from the digits [4,5,7,[11], [12], [13], [14], [15], [16]]. Though classically originating from veins, five cases have been reported where the lesions arose from the digital artery [7,11,13,16,17]. Two cases were extraordinary because the lesion originated from the palmar arterial arch [18,19]. Moreover, two other cases described angioleiomyomas arising from the distal ulnar artery, one of which coexisted with symptoms of superficial sensory branch of the ulnar nerve compression [20,21]. Sporadic conditions were reported in which the tumor had involved the hand’s bones or tendons [12,15]. In a report, a horseshoe-shaped angioleiomyoma was found encroaching on the palmar shaft of the proximal phalanx of the right index beneath the flexor tendon. Although significantly depressed, but intact, cortex was noted intraoperatively, the patient’s day-to-day function was not negatively impacted for the two-year follow-up duration [12]. In another case, a mass on the left thumb was found below the extensor pollicis longus breaching the cortical bone and invading the medullary canal. The patient was back to baseline condition within a year [15].

Treatment by local excision is sufficient, as described in this paper; On the same manner, simple wound closure is also sufficient. However, Houdek et al. (2013) performed a wide local excision on the premise of high preoperative suspicion of an undifferentiated pleomorphic sarcoma. Likewise, they reported different methods of wound closure in two of their twenty-four cases. A patient required a local, proximally based rotational flap in order to cover the fifth digit while the other underwent split-thickness skin grafting of the index finger [1]. further reported surgical methods include wide local excision and ray amputation of the digit in case of recurrent malignancies [4,5] and vascular grafting or repair when the digital artery is involved with poor collateral supply [11]. In a case of angioleiomyoma involving the radial digital artery, removal of the mass with end-to-end microsurgical anastomosis was done [17]. A similar approach was followed in a case involving the distal ulnar artery [21]. It was recommended that Allen’s test should be performed and atraumatic vascular clamp should be placed over the involved artery with tourniquet deflation to inspect the adequacy of the collateral supply [11]. In case the tumor arises from a non-patent digital artery, excision *in toto* with segmental resection of the involved artery could be sufficient, as described by Robinson and Kalish [16]. In this paper, however, there was no need for vascular grafting or reconstruction.

Five cases of recurrence have been published with the maximum point of recurrence in time being seven years postoperatively [8]. A peculiar observation, in a case of recurrence accompanied by malignant transformation to leiomyosarcoma, was that the biopsy demonstrated a greater degree of cellularity, in contrast to the typical acellular angioleiomyoma. henceforth, the authors of that report recommended following-up the cases that show a similar pattern on biopsy more closely. No further data about the patient was reported as she lost follow-up after three years post-operatively [5]. Another case involving the pulp of the index finger revealed a similar finding on histopathology. The mass recurred few weeks following excisional biopsy, which prompted for another procedure. Finally, it recurred rapidly after the second operation with an explosive exophytic growth and bleeding which necessitated ray amputation. There was no metastasis. The patient lost follow-up after four years [4]. Complications rarely occur. Post-operative hematoma [1], malignant transformation [4,5] and bone erosion were reported in the literature.

## 4 Conclusion

Many different tumors may arise from the hand. An uncommon subset of which is the benign angioleiomyoma. Contrary to typical belief, its origin maybe arterial, adding to its rarity. It can present as a painless or painful, firm swelling in middle-aged ladies, characters that do not contribute to differentiation from other conditions. To complicate matters more, imaging modalities provide limited insight into the nature of the lesion. Histological studies remain the cornerstone of diagnosis. Local excision should suffice in most of the conditions. This paper reports a case of a typical angioleiomyoma of the hand, yet rare in its origin, from the ulnar digital artery. The treating surgeon should consider this condition in the differential diagnosis when encountered.

## Conflicts of interest

None.

## Funding

None.

## Ethical approval

Exempted from the IRB approval.

## Consent

Written informed consent was obtained from the patient for publication of this case report and accompanying images. A copy of the written consent is available for review by the Editor-in-Chief of this journal on request.

## Author contribution

1. Abdullah Kattan; Supervision; Treatment of the patient and manuscript editing.
2. Khalid Arab: Manuscript editing.
3. Mohammed Alswayyed: Manuscript editing.
4. Emran Al Gadiem: Manuscript editing.
5. Qutaiba Shah Mardan: Literature review and overall writing of the manuscript.

## Registration of research studies

This paper does not require registry as it is a case report about a condition that has been discussed in prior papers. There is no additional harm to the patient nor an intervention is being applied on the patient.

## Guarantor

Abdullah Kattan.

Qutaiba Shah Mardan.

## Provenance and peer review

Not commissioned, externally peer-reviewed.
